# A Key Gene, *PLIN1*, Can Affect Porcine Intramuscular Fat Content Based on Transcriptome Analysis

**DOI:** 10.3390/genes9040194

**Published:** 2018-04-04

**Authors:** Bojiang Li, Qiannan Weng, Chao Dong, Zengkai Zhang, Rongyang Li, Jingge Liu, Aiwen Jiang, Qifa Li, Chao Jia, Wangjun Wu, Honglin Liu

**Affiliations:** Department of Animal Genetics, Breeding and Reproduction, College of Animal Science and Technology, Nanjing Agricultural University, 210095 Nanjing, China; 2015205004@njau.edu.cn (B.L.); wqn9134@163.com (Q.W.); 2016105025@njau.edu.cn (C.D.); 2017105031@njau.edu.cn (Z.Z.); 2015105013@njau.edu.cn (R.L.); 2014105014@njau.edu.cn (J.L.); 671218l@sina.com (A.J.); liqifa@njau.edu.cn (Q.L.); jiachao@njau.edu.cn (C.J.)

**Keywords:** pig, intramuscular fat content, transcriptome, candidate genes, *PLIN1*

## Abstract

Intramuscular fat (IMF) content is an important indicator for meat quality evaluation. However, the key genes and molecular regulatory mechanisms affecting IMF deposition remain unclear. In the present study, we identified 75 differentially expressed genes (DEGs) between the higher (H) and lower (L) IMF content of pigs using transcriptome analysis, of which 27 were upregulated and 48 were downregulated. Notably, Kyoto Encyclopedia of Genes and Genomes (KEGG) enrichment analysis indicated that the DEG perilipin-1 (*PLIN1*) was significantly enriched in the fat metabolism-related peroxisome proliferator-activated receptor (PPAR) signaling pathway. Furthermore, we determined the expression patterns and functional role of porcine *PLIN1.* Our results indicate that *PLIN1* was highly expressed in porcine adipose tissue, and its expression level was significantly higher in the H IMF content group when compared with the L IMF content group, and expression was increased during adipocyte differentiation. Additionally, our results confirm that *PLIN1* knockdown decreases the triglyceride (TG) level and lipid droplet (LD) size in porcine adipocytes. Overall, our data identify novel candidate genes affecting IMF content and provide new insight into *PLIN1* in porcine IMF deposition and adipocyte differentiation.

## 1. Introduction

Intramuscular fat (IMF), corresponding to pork marbling, refers to the fat located within muscles, which is associated with the number and size of intramuscular adipocytes [1]. Previous studies have indicated that IMF is closely related to flavor, juiciness, and tenderness [2,3]; lower IMF content results in dry and less-flavorful meat, and IMF content is positively associated with sensory quality traits of meat and consumer acceptability [4]. When IMF content increases from 1 to 3%, sensory quality is significantly improved [5]. In the past few decades, breeders have focused on lean meat yield and backfat thickness, which result in decreased IMF content [6,7]. At present, the average IMF content of longissimus dorsi muscle is about 2% in many lean pig breeds [8,9,10]. A previous study indicated that consumer preference for appearance in pork is strongly associated with IMF content [11], therefore improving IMF content has become an important objective in modern pig breeding programs [12]. Although the heritability of IMF content is relatively high, varying from 0.21 to 0.86, it is difficult to determine the IMF content in livestock, resulting in its rare use in pig breeding programs [1]. Therefore, developing of molecular breeding technologies could provide a valid way to improve the IMF content in pigs.

In recent years, many quantitative trait loci (QTL) and candidate genes affecting IMF content have been identified [3,13,14,15,16]. To date, 25,610 QTLs representing 646 different traits have been curated in a pig QTL database (PigQTLdb). Among these QTLs, 105 are associated with intramuscular fat content (https://www.animalgenome.org/cgi-bin/QTLdb/SS/index, [17], release 34, 21 December 2017). Multiple differentially expressed genes (DEGs) related to IMF content were identified in the pig using microarray technology, including acetyl-coenzyme A acyltransferase 1 (*ACAA1*), lipoprotein lipase (LPL), hormone-sensitive lipase (HLS), fatty acid binding protein 3 (*FABP3*), and FLJ36031 (*pFLJ*) genes [18]. A genome-wide association study (GWAS) demonstrated that a mutation (C > A) located in intron 9 of phosphorylase kinase catalytic subunit gamma 1 *(PHKG1)* can affect its expression and is associated with meat quality traits, including IMF content [19]. Moreover, stearoyl CoA desaturase (*SCD*) and leptin (*LEP*) genes were upregulated in semimembranosus muscle with high IMF content using transcriptome analysis [20]. Melanocortin 4 receptor (*MC4R*), mitochondrial phosphoenolpyruvate carboxykinase (*PEPCK*), and *SCD* genes expression levels are significantly correlated with IMF content in Duroc × Shanzhu commercial crossbred pigs [16]. However, whether these are the key genes affecting IMF content remains ambiguous. Moreover, some results are not consistent, due to the differences in genetic background, technologies, and breed, etc. As biological technology has developed, high-throughput sequencing has become the efficient and convenient method to identify candidate genes influencing IMF.

Herein, we used RNA sequencing (RNA-Seq) technology to identify the novel candidate genes affecting pork intramuscular fat deposition. We further demonstrated that the candidate gene, *PLIN1*, is involved in the regulation of IMF content in pigs, and can regulate triglyceride (TG) level and lipid droplet (LD) size in porcine adipocytes. Overall, this study provides valuable information for identifying novel candidate genes affecting IMF content, and clarifies the molecular mechanism of candidate gene *PLIN1* affecting IMF content.

## 2. Materials and Methods

### 2.1. Ethics Statement

All experiments were undertaken in accordance with the guidelines of the regional Animal Ethics Committee and were approved by the Institutional Animal Care and Use Committee of Nanjing Agricultural University (SYXK2011-0036, 6 December 2011).

### 2.2. Animals, Samples Collection and Phenotypes Measurement

Experimental pigs used in this study were from 279 commercial hybrid pigs (Pietrain × Duroc) × (Landrace × Yorkshire), 194 castrated males and 85 females, and were provided by Guangdong Wen’s Foodstuffs Group Co., Ltd (Huaian, China), which were raised under standard conditions and fed ad libitum with free access to water. Experimental pigs were slaughtered at an average age of 170 days in 11 random batches (Nos. 1–11). Samples of longissimus dorsi muscle, liver, spleen, kidney, heart, and adipose tissue were collected, snap-frozen in liquid nitrogen, and stored at −80 °C until RNA isolation. Intramuscular fat (IMF) content of longissimus dorsi muscles of 279 pigs was measured using the Soxhlet extraction method, as described previously [21]. 

### 2.3. RNA Extraction, Library Preparation and Sequencing

A total of 18 longissimus dorsi samples were used to perform the RNA-Seq, which were derived from 3 batches (Nos. 2, 3, and 5) randomly selected from 11 batches; three individuals with higher (H) (*n* = 3) and lower (L) (*n* = 3) IMF content were picked out from each batch. Total RNA was extracted using TRIzol (Invitrogen, Carlsbad, CA, USA) according to the manufacturer’s protocol, and RNA concentration and integrity were evaluated by an Agilent 2100 Bioanalyzer (Agilent Technologies, Santa Clara, CA, USA). Subsequently, an equal amount of RNA from 3 samples of each batch (3 with H IMF content and 3 with L IMF content) were pooled to generate one sequencing library. A total of 6 libraries were constructed using the NEBNext^®^ Ultra™ RNA Library Prep Kit for Illumina (NEB, Ipswich, MA, USA), following the manufacturer’s instructions. Size, purity, and concentration of the sample library were evaluated by Agilent 2100 Bioanalyzer (Agilent Technologies, Santa Clara, CA, USA), and sequencing was performed on an Illumina HiSeq 4000 (Illumina, San Diego, CA, USA) to produce 150 bp paired-end reads. All the RNA-Seq data related to this study have been deposited in Sequence Read Archive (SRA, National Institute of Health, Bethesda, MD, USA) with the accession code SRP125462.

### 2.4. Raw Data Filtering and Transcript Analysis 

High-quality clean reads were obtained by removing low-quality (the percentage of low-quality bases of quality value ≤ 15 is more than 50% in a read), adapter-polluted, and high content of unknown base (N) reads (more than 5%) from raw reads. Then, clean reads were aligned to the pig reference genome (Sscrofa10.2) using HISAT [22]. Sample transcripts were reconstructed using StringTie [23], then reconstructed transcripts were compared to the reference annotation using Cuffcompare [24]. Coding potential of novel transcripts was predicted using the Coding Potential Calculator (CPC) [25]. Then, coded novel transcripts were merged with reference transcripts to obtain a complete reference, and downstream analysis was based on this reference.

### 2.5. Screening of Differentially Expressed Genes, and Gene Ontology and Kyoto Encyclopedia of Genes and Genomes Analysis

Clean reads were mapped to reference using Bowtie2 [26], and then gene expression levels were calculated with RSEM [27] and described by fragments per kilobase of exon per million fragments mapped values. Differentially expressed genes between two groups were determined with NOIseq [28], and the parameters were set to log2 (fold change) ≥ 1.00 and probability ≥ 0.7. To further understand the function of DEGs, Gene Ontology (GO) analysis was performed using the Blast2GO program [29]. Kyoto Encyclopedia of Genes and Genomes (KEGG) pathway annotation was conducted using the BLASTX algorithm against the KEGG database (http://www.genome.jp/kegg/, Kanehisa Laboratories, Kyoto, Japan). Functional enrichment of GO and KEGG was performed using the OmicShare tools, a free online platform for data analysis (http://www.omicshare.com/tools). Corrected *p*-value ≤ 0.05 was defined as significantly enriched. 

### 2.6. Preadipocytes Isolation, Culture, and Differentiation

Porcine preadipocytes were isolated from longissimus dorsi muscle of 3-day-old piglets, as previously described [30]. Cells were cultured in DMEM/F12 (Gibco, Waltham, MA, USA) supplemented with 10% fetal bovine serum (FBS) (Gibco, Waltham, MA, USA), penicillin (100 U/mL) (Gibco, Waltham, MA, USA), and streptomycin (100 μg/mL) (Gibco, Waltham, MA, USA) at 37 °C in 5% CO_2_. To induce differentiation into mature adipocytes, the medium was changed by DMEM/F12 (Gibco, Waltham, MA, USA) containing with 10% FBS, 0.1 μM dexamethasone, 0.5 mM 3-isobutyl-1-methylxanthine, and 5 μg/mL insulin after cells reached 90% confluence.

### 2.7. PLIN1 Knockdown

For knockdown of *PLIN1*, small interfering RNA (siRNA) was purchased from GenePharma (Shanghai, China). The sequences for siRNA were the following: *siControl*, sense: 5′-UUCUCCGAACGUGUCACGUTT-3′ and antisense: 5′-ACGUGACACGUUCGGAGAATT-3′; si*PLIN1*, sense: 5′-CCAUCUCUCAACACACCUUTT-3′ and antisense: 5′-AAGGUGUGUUGAGAGAUGGTT-3′. Adipocytes were transfected with 75 pmol siRNA by Lipofectamine^®^ 3000 reagent (ThermoFisher Scientific, Waltham, MA, USA) on the third day following the induction of differentiation. Forty-eight hours after transfection, cells were used to extract RNA and protein, and to measure TG level and image lipid droplet size.

### 2.8. Quantitative Reverse-Transcription PCR Analysis

Total RNA from longissimus dorsi muscle, other tissues (liver, spleen, kidney, heart, and adipose), and cells were extracted using TRIzol (Invitrogen, Carlsbad, CA, USA), and then reverse transcribed into complementary DNA (cDNA) using Primescript RT Master Kit (Takara, Dalian, China) according to the manufacturer’s instructions. Primers of DEGs were designed by Primer 3 (http://bioinfo.ut.ee/primer3/) [31,32] and are listed in Table S1. Real-time quantitative reverse transcription polymerase chain reaction (qRT-PCR) was performed with AceQ qPCR SYBR Green Master Mix (Vazyme, Nanjing, China) in a reaction volume of 20 μL. The cycling parameters were as follows: 95 °C for 5 min, followed by 40 amplification cycles, each at 95 °C for 10 s, then 60 °C for 30 s. All reactions were performed in triplicate for each sample. The glyceraldehyde-3-phosphate dehydrogenase (*GAPDH)* gene was used as internal control to normalize the relative expression of genes. The gene expression levels were calculated by the ΔΔ*C*t value method [33].

### 2.9. Western Blotting

Longissimus dorsi muscle from pig cells were lysed with RIPA lysis buffer (Beyotime, Nanjing, China) supplemented with 1% protease inhibitor cocktail (Selleck, Houston, TX, USA). Protein concentration was measured using bicinchoninic acid (BCA) Protein Assay Kit (Beyotime, Nanjing, China). An equal amount of protein was run on sodium dodecyl sulfate polyacrylamide gel electrophoresis (SDS-PAGE), then transferred to polyvinylidene difluoride (PVDF) membranes (Millipore, Billerica, MA, USA). The membranes were blocked with 5% bovine serum albumin (BSA) (Sigma, St Louis, MO, USA) for 1 h at room temperature and incubated with rabbit polyclonal to anti-pig PLIN1 antibody (1:1000) (Abcam, Cambridge, MA, USA) or GAPDH antibody (1:1000) (Abcam, Cambridge, MA, USA) at 4 °C overnight. Next, the membranes were incubated with HRP–conjugated goat anti-rabbit secondary antibody (1:2000) (Abcam, Cambridge, MA, USA) for 1 h at room temperature. The signals were detected with Pierce Fast Western Blot Kit (ThermoFisher Scientific, Waltham, MA, USA) on a chemiluminescence detection system (LAS-4000 Imager, Tokyo, Japan). The band intensities were quantified using ImageJ 1.42q software (National Institutes of Health, Bethesda, MD, USA), and the level of GAPDH was used to normalize the expression of the target protein.

### 2.10. Immunohistochemistry

Longissimus dorsi muscle was fixed in 4% paraformaldehyde (PFA) (Beyotime, Nanjing, China) overnight at 4 °C, embedded in paraffin, and cut with a microtome onto glass slides (Beyotime, Nanjing, China). Sections were blocked with 1% BSA/PBS, incubated with rabbit polyclonal to anti-pig PLIN1 antibody at 1:100 dilution. Slides were incubated with Cy3—conjugated goat anti-rabbit secondary antibody (1:100 dilution) (Abcam, Cambridge, MA, USA), washed three times with phosphate-buffered saline (PBS) (Gibco, Waltham, MA, USA), stained with 40′,6-diamidino-2-phenylindole (DAPI) (Beyotime, Nanjing, China), and examined by fluorescence microscopy.

### 2.11. Immunofluorescence Staining

Adipocyte cells were washed twice with PBS, fixed in 4% PFA for 30 min, and permeabilized in 0.5% Triton X-100 (Beyotime, Nanjing, China) for 20 min. Cells were then blocked with 1% BSA in PBS for 1 h, incubated with rabbit polyclonal to anti-pig PLIN1 antibody (1:100) in 1% BSA/PBS for 2 h, washed three times, then incubated with fluorescently labeled goat anti-rabbit secondary antibody (1:200) (Abcam, Cambridge, MA, USA) in 1% BSA/PBS for 1 h. Cells were washed three times with PBS, incubated with Bodipy (ThermoFisher Scientific, Waltham, MA, USA) and DAPI for 10 min, and imaged by confocal microscopy.

### 2.12. Bodipy Staining

To stain for neutral lipids of porcine adipocytes, cells were washed twice with PBS, fixed in 4% PFA for 30 min, incubated with Bodipy (1:1000) for 10 min, and imaged by confocal microscopy.

### 2.13. Cellular Triglyceride Determination

For cellular TG level measurement, adipocyte cells were lysed with cell lysis buffer (Nanjing Jiancheng, Nanjing, China). Protein concentrations in extracts were measured by BCA Protein Assay Kit. Then, TG levels were determined by TG determination kit (Nanjing Jiancheng, Nanjing, China) according to the manufacturer’s protocols. TG levels were normalized to the protein concentration of each sample.

### 2.14. Statistical Analyses

Statistical analyses were conducted using SPSS 20.0 (SPSS Inc., Chicago, IL, USA). Statistical significance of the difference between two means was calculated using Student’s *t* test or ANOVA test. A value of *p* < 0.05 was considered to represent a statistically significant difference.

## 3. Results

### 3.1. Phenotypic Variations between Extreme Groups

In this study, we measured the IMF content of longissimus dorsi muscle of 279 pigs in 11 batches. The values of mean, standard deviation (SD), minimum, maximum, and coefficient of variation (CV) of IMF content for each group and all groups are shown in Table S2. The results show that the mean and standard deviation of IMF content for the entire pig population were 2.64% and 0.91%, respectively. Moreover, IMF content maximum and minimum were 6.56% and 1.09%, respectively. To reduce the effect of intergroup differences in the RNA-Seq, we randomly chose three groups from 11 batches, and picked out three extremely high and three extremely low IMF content individuals from each group, and mixed RNA from three individuals equally to generate a pool. A total of six libraries were constructed (Figure S1), which were named H2, H3, H5, L2, L3, and L5. Statistical analysis showed that the IMF content between higher (H) and lower (L) in each group was significantly different (*p* < 0.01; Table 1), while carcass weight was not different between the H and L IMF groups.

### 3.2. Summary of RNA Sequencing Data

Output of RNA-Seq data is shown in Table 2. After filtering low-quality, adapter-polluted, and high content of unknown base (N) reads, we acquired 268,826,758 clean reads (from 44,222,498 to 45,247,560 for each sample) and 40.33 giga (G) clean bases (from 6.63 G to 6.79 G for each sample) in six libraries. The average quality value of Q20 (Phred quality score > 20 and an error rate < 0.01) was 94.08% for each library. An average of approximately 65% of clean reads were mapped to the pig reference genome, in which 58.80% were uniquely mapped to the reference genome (Table 2). Moreover, we identified 13,568 novel transcripts, which contained 9628 coding transcripts and 3940 noncoding transcripts.

### 3.3. Gene Expression Analysis, and Differentially Expressed Genes Identification

**Figure 1:**
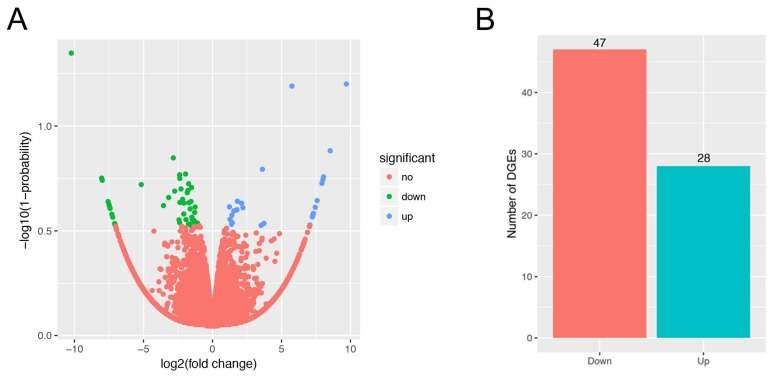
Identification of differentially expressed genes (DEGs). (**A**) Volcano plot showing DEGs in higher (H) and lower (L) groups. The *x* axis represents the value of log2 (L/H), and the *y* axis represents the value of −log10 (1-probability). Red, green, and blue dots represent unchanged, downregulated, and upregulated DEGs between the H and L groups; (**B**) Statistics of DEGs between the H and L groups. The *x* axis represents downregulated and upregulated genes, and the *y* axis represents the number of DEGs.

**Figure 2:**
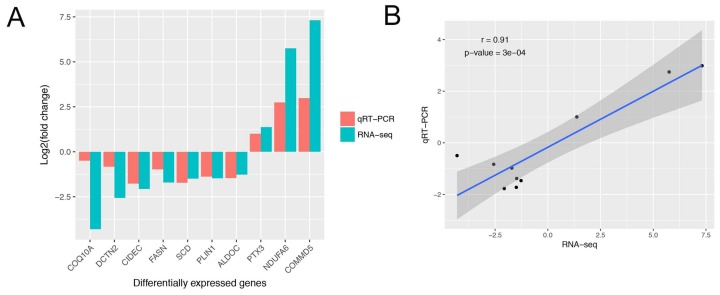
Real time quantitative reverse-transcription polymerase chain reaction (qRT-PCR) validation of differentially expressed genes (DEGs) and correlation with RNA sequencing (RNA-Seq). (**A**) Validation of the DEGs by qRT-PCR (*n* = 3). The *x* axis represents DEGs and the *y* axis represents the log2 (L/H) for qRT-PCR and RNA-Seq; (**B**) Correlation analysis of DEGs between qRT-PCR and RNA-Seq. The *x* and *y* axes represent the log2 (L/H) measured by RNA-Seq and qRT-PCR, respectively.

Expression levels of all genes in the six sequencing libraries are listed in Table S3. A total of 16,798 genes were identified in all samples, and number of genes of each sample ranged from 13,851 to 14,686. To identify candidate genes affecting IMF content, we used NOIseq software to detect DEGs between the H and L IMF content groups. A total of 75 DEGs were identified, 28 upregulated and 47 downregulated genes (Figure 1). Detailed information for all DEGs is shown in Table S4. To validate the RNA-Seq results, 10 DEGs (coenzyme Q10A (*COQ10A*), dynactin subunit 2 (*DCTN2*), cell death inducing DFFA like effector c (*CIDEC*), fatty acid synthase (*FASN*), *SCD*, *PLIN1*, aldolase C fructose-bisphosphate (*ALDOC*), pentraxin 3 (*PTX3*), NADH ubiquinone oxidoreductase subunit A6 (*NDUFA6*), and COMM domain-containing protein 5 (*COMMD5*)) were randomly selected to examine the gene expression level using qRT-PCR. The results showed that expression patterns of these genes from qRT-PCR were consistent with RNA-Seq (Figure 2A), and the correlation of the two methods was relatively high, with a correlation coefficient of 0.91 (Figure 2B), suggesting that DEGs identified from RNA-Seq were reliable in this study.

### 3.4. Gene Ontology and Kyoto Encyclopedia of Genes and Genomes Enrichment Analysis of Differentially Expressed Genes

**Figure 3:**
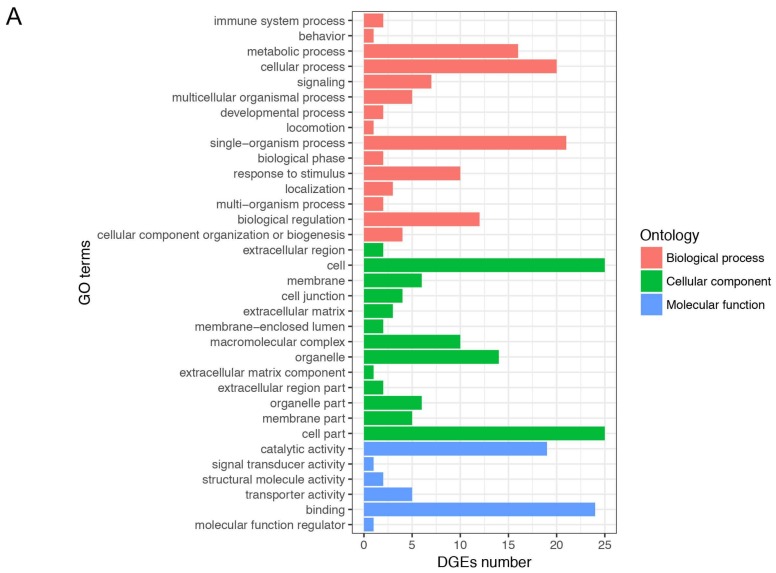

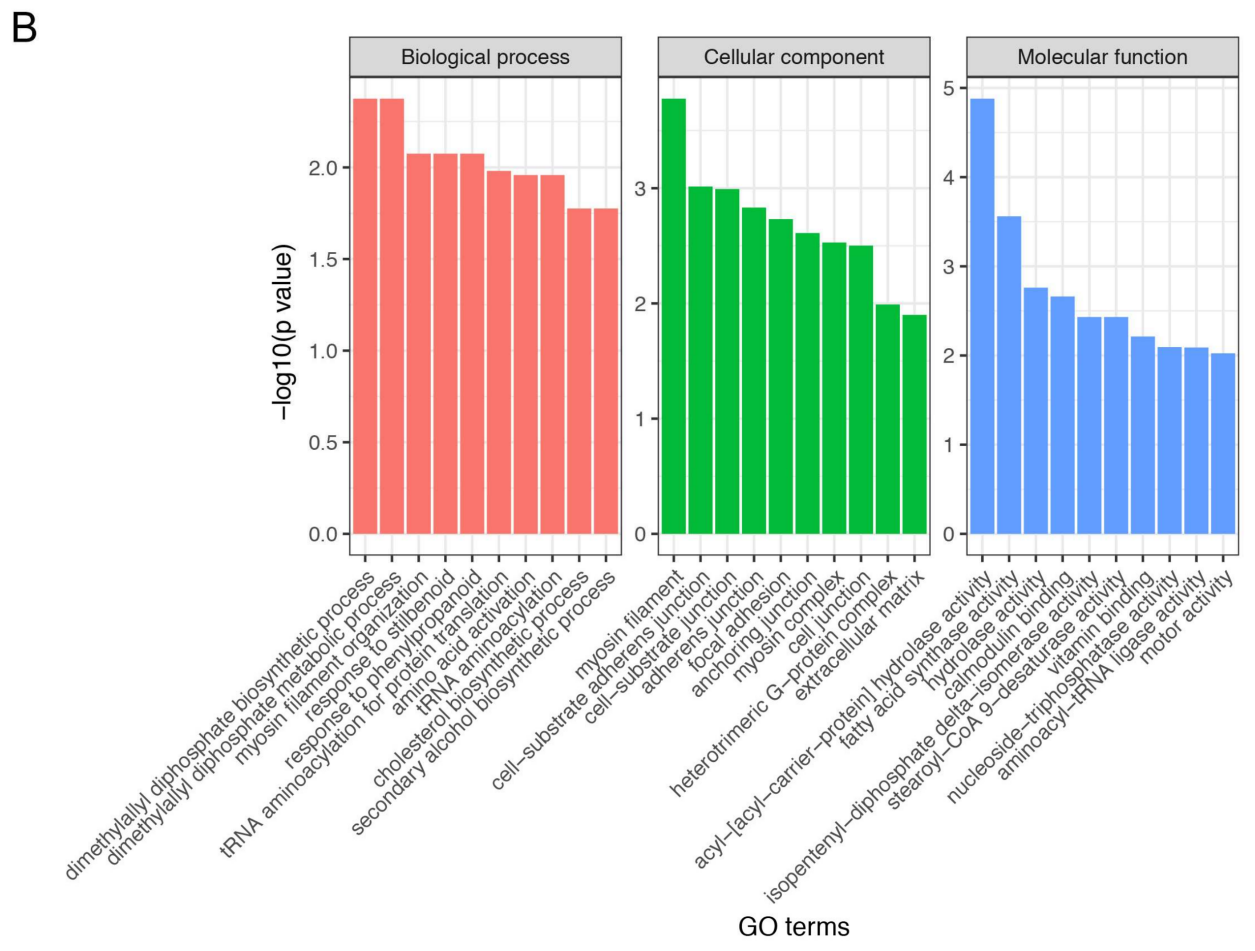
Gene Ontology (GO) analysis of differentially expressed genes (DEGs). (**A**) GO categories of DEGs. The *x* axis represents number of DEGs and the *y* axis represents GO terms; (**B**) The most enriched 10 GO terms in biological process, cellular component, and molecular function. The *x* axis represents GO terms and the *y* axis represents the value of −log10 (*p*-value).

**Figure 4:**
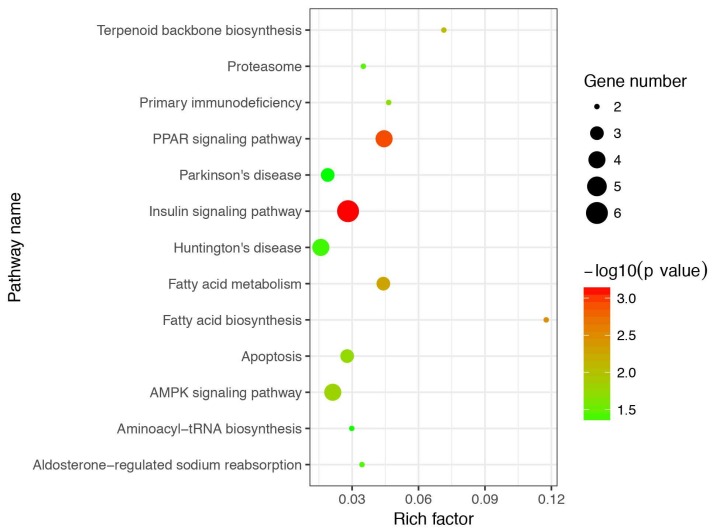
Kyoto Encyclopedia of Genes and Genomes (KEGG) enrichment analysis of differentially expressed genes (DEGs). The *x* axis represents rich factor and the *y* axis represents pathway. Size and color of the bubble represent the amount of differentially expressed genes enriched in pathway and enrichment significance, respectively.

To clarify the functional classification of DEGs in the regulation process of IMF, we performed GO analysis for DEGs. All GO terms of DEGs were summarized into three main GO categories, biological process, cellular component and molecular function, and 34 subcategories (Figure 3A). Among these subcategories, the “cell” and “cell part” belonging to the cellular component contained the most DEGs. The enriched analysis results showed that 27, 27, and 39 GO terms were significantly enriched in biological process, cellular component, and molecular function, respectively (Table S5 and Figure 3B). In order to elucidate the biological pathways of DEGs, we further performed pathway enrichment analysis, and detailed information on pathway enrichment is listed in Table S6. In total, 13 pathways were significantly enriched (Figure 4).

### 3.5. Association of PLIN1 Expression Level with Porcine Intramuscular Fat Content

**Figure 5:**
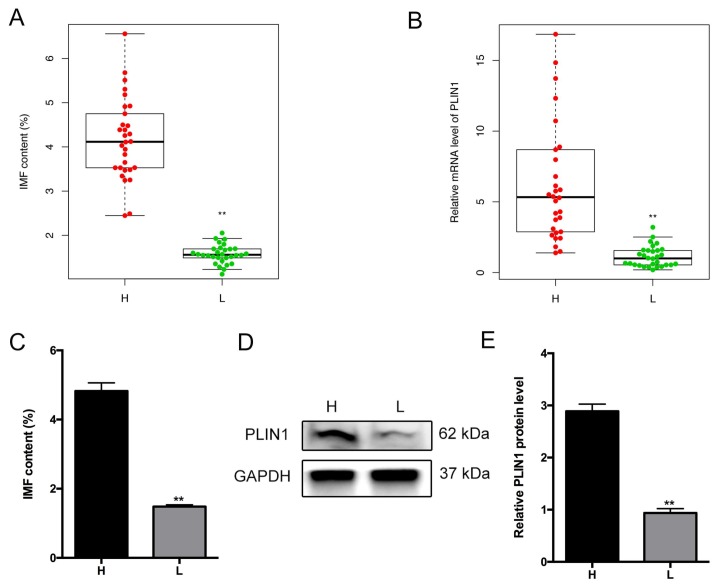
Expression level of *PLIN1* gene between higher (H) and lower (L) intramuscular fat (IMF) groups. (**A**) IMF content; and (**B**) Real-time quantitative reverse transcription polymerase chain reaction (qRT-PCR) analysis of *PLIN1* messenger RNA (mRNA) expression levels between H and L IMF groups (*n* = 30). ** *p* < 0.01; (**C**) IMF content; and (**D**,**E**) Western blotting analysis for *PLIN1* protein expression levels between H and L IMF groups (*n* = 6).

Previous research indicated that the peroxisome proliferator-activated receptor (PPAR) signaling pathway has an important role in adipogenesis [34]. Intriguingly, the DEG *PLIN1* was classified in the PPAR signaling pathway, which attracted our attention in the current study. Moreover, previous study has shown that *PLIN1* is a lipid droplet protein and plays an important role in the lipolysis [35]. In addition, *PLIN1* was chosen as an important candidate gene affecting IMF according to its expression abundance (medium), fold change (>2.0), and probability (>0.70). To demonstrate the association of *PLIN1* expression level with IMF content in pigs, we detected *PLIN1* messenger RNA (mRNA) and protein levels between the H and L IMF content groups. First, we examined the *PLIN1* mRNA levels in 60 samples, 30 higher IMF individuals and 30 lower IMF individuals, and IMF content was significantly different between the two groups (*p* < 0.01) (Figure 5A). Correspondingly, the mRNA level of *PLIN1* was significantly higher in the H IMF content group than in the L IMF content group (*p* < 0.01) (Figure 5B). We further confirmed that the *PLIN1* protein level was significantly increased (*p* < 0.01) in the H IMF content group when compared to the L IMF content group (Figure 5C–E).

### 3.6. PLIN1 Is Induced in the Process of Adipocyte Differentiation

**Figure 6:**
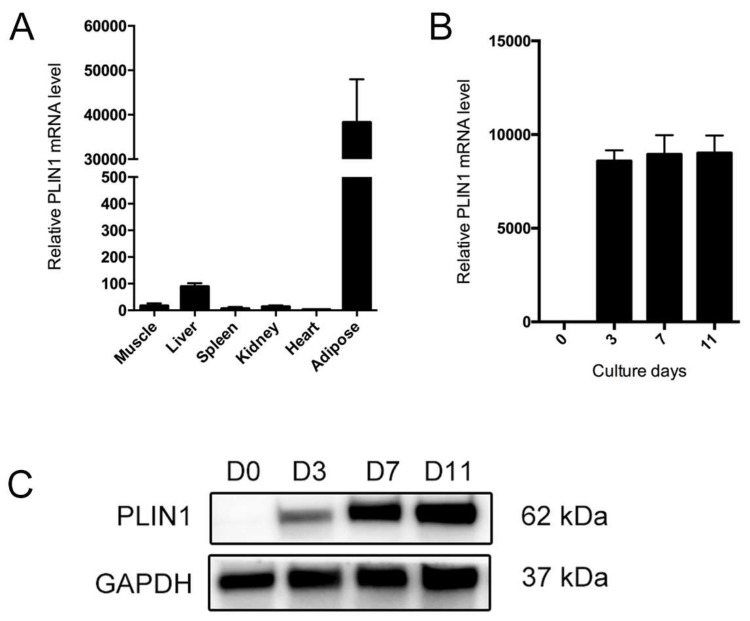
Expression patterns of *PLIN1* gene in different tissues and stages of adipocyte differentiation. (**A**) Real-time quantitative reverse transcription polymerase chain reaction (qRT-PCR) analysis for *PLIN1* messenger RNA (mRNA) expression levels in porcine adipose and other tissues; (**B**) qRT-PCR analysis for *PLIN1* Mrna; and (**C**) Western blotting analysis for *PLIN1* protein expression levels at 0, 3, 7, and 11 d after adipocyte differentiation. All data are presented as mean ± standard error (SE); *n* = 3.

To verify the relationship of *PLIN1* with adipocyte differentiation, we detected the *PLIN1* expression levels in different tissues and stages of adipocyte differentiation. Our results indicate that the *PLIN1* gene was highly expressed in adipose tissue compared with other tissues (Figure 6A). The expression of *PLIN1* at mRNA and protein levels was significantly upregulated at 3, 7, and 11 days after induction of differentiation, while *PLIN1* was not expressed in the preadipocytes (Figure 6B,C).

### 3.7. Location of PLIN1 in the Longissimus Dorsi Muscle and Adipocytes

**Figure 7:**
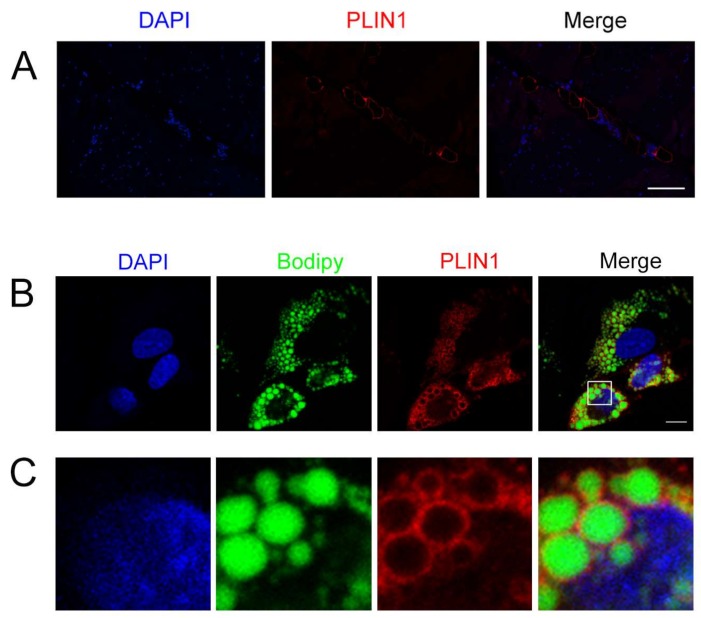
*PLIN1* localized in porcine longissimus dorsi muscle and adipocytes. (**A**) Immunohistochemistry of *PLIN1* in porcine longissimus dorsi muscle. *PLIN1* was stained with antibody PLIN1 (red). Nuclei were stained with 4′,6-diamidino-2-phenylindole (DAPI, blue). Scale bars: 100 μm; (**B**,**C**) *PLIN1* immunofluorescence at the surface of lipid droplets (LDs) in porcine adipocytes. *PLIN1* was stained with antibody PLIN1 (red). Lipid droplets were stained with Bodipy (green). Nuclei were stained with DAPI (blue). Scale bars: 10 μm.

To confirm the location of *PLIN1* in longissimus dorsi muscle of pigs, we conducted immunohistochemistry. The results show that porcine *PLIN1* was localized in adipocytes of porcine longissimus muscle (Figure 7A). Furthermore, the location of *PLIN1* was examined in adipocytes of pigs using immunofluorescence staining, and it was localized around lipid droplets stained with Bodipy (Figure 7B,C).

### 3.8. PLIN1 Knockdown Decreases Triglyceride Level and Lipid Droplet Sizes in Pig Adipocytes

**Figure 8:**
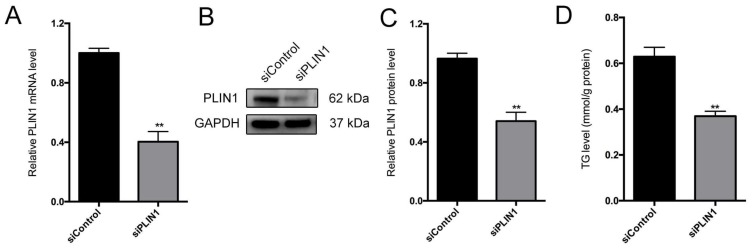

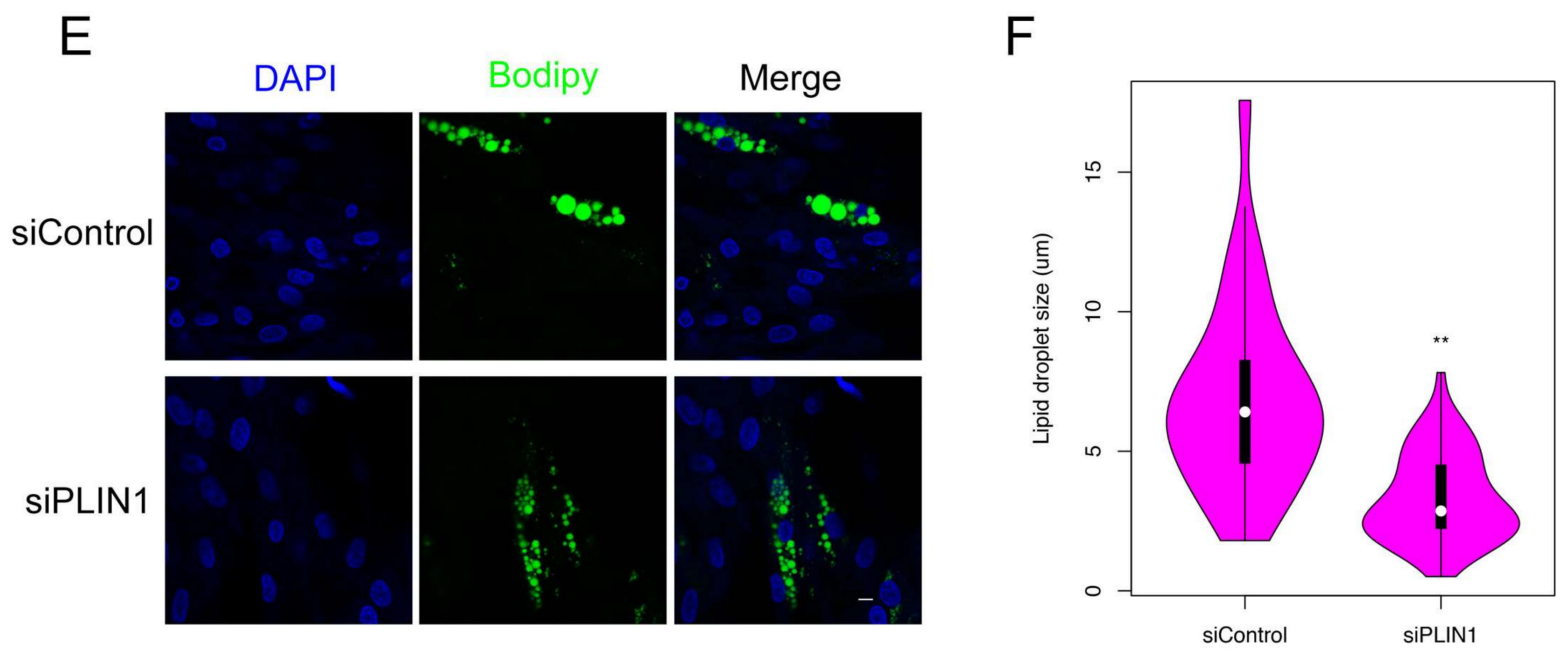
*PLIN1* knockdown resulted in decreased triglyceride (TG) level and lipid droplet(LD) size. (**A**) Real-time quantitative reverse transcription polymerase chain reaction (qRT-PCR) analysis for *PLIN1* messenger RNA (mRNA); (**B**,**C**) Western blotting analysis of *PLIN1* protein expression in porcine adipocytes transfected with *siControl* or *siPLIN1* after 48 h; (**D**) Measurement of TG level in porcine adipocytes transfected with *siControl* or *siPLIN1* after 48 h; (**E**) Representative images showing morphology; and (**F**) vioplot showing statistical analysis of LD size in porcine adipocytes transfected with *siControl* or *siPLIN1* after 48 h. Lipid droplets were stained with Bodipy (green). Nuclei were stained with DAPI (blue). Scale bars: 10 μm. All data are presented as mean ± SE, ** *p* < 0.01.

To confirm the molecular mechanisms of *PLIN1* in lipid droplets formation and TG level, *PLIN1* was knocked down in adipocytes using siRNA. We first detected the efficiency of *PLIN1* depletion in pig adipocytes, and data indicated that mRNA and protein levels of *PLIN1* were markedly decreased in the *PLIN1* knockdown group compared to control (Figure 8A–C). Triglyceride level was significantly reduced in *PLIN1* knockdown adipocytes (Figure 8D). Moreover, knockdown of *PLIN1* resulted in a significant decrease of lipid droplet size in pig adipocytes (Figure 8E,F).

## 4. Discussion

Intramuscular fat content is significantly related to the sensory quality of cooked meat, such as juiciness and tenderness [36]. The range of measured IMF content of longissimus dorsi muscle derived from 279 pigs was large. Therefore, extremely higher and lower IMF content groups are suitable for identifying the novel candidate genes affecting IMF content by RNA-Seq analysis.

A total of 13,568 novel transcripts were obtained in the study, which was consistent with the number of novel transcripts obtained from porcine digitorum longus and soleus muscles (10,962 and 9686, respectively) [37]. Furthermore, a previous study showed that 18,959 genes were detected in biceps femoris and soleus muscles [38]. Our results also indicated that the number of genes from longissimus dorsi muscle was similar to that from other muscles.

Moreover, GO enrichment analysis results indicated that most significant GO terms, including “acyl-(acyl-carrier-protein) hydrolase activity”, “fatty acid synthase activity”, and “myosin filament”, may play an important role in IMF deposition. Interestingly, acyl-(acyl–carrier-protein) hydrolase activity and fatty acid synthase activity contained the differentially expressed gene *FASN*. The *FASN* gene plays a crucial role in the process of lipogenesis, and it is a large multienzyme involved in all steps of fatty acid synthesis in mammals [39]. *FASN* synthesizes long-chain fatty acids by using acetyl–CoA as a primer, malonyl–CoA as a two-carbon donor, and nicotinamide adenine dinucleotide phosphate (NADPH) as a reducing equivalent [40]. The mRNA abundance of *FASN* has a significant positive correlation with IMF content in longissimus dorsi muscle of Korean cattle [41]. Zhu et al. reported that *FASN* located at 51.3 Mb on *Bos taurus* autosome 19 (BTA19) is related to fatty acid composition using high-density single nucleotide polymorphism (SNP) array in Chinese Simmental beef cattle [42]. In the present study, *FASN* expression level was associated with IMF content, which was consistent with previous results [43].

Furthermore, we observed several significantly enriched pathways, including insulin signaling pathway, PPAR signaling pathway, fatty acid biosynthesis, fatty acid metabolism, and terpenoid backbone biosynthesis, suggesting that DEGs in these pathways may be related to IMF biosynthesis and metabolism. DEGs in these pathways, such as *FASN*, *SCD*, *PLIN1*, 3-phosphoinositide dependent protein kinase 1 (*PDK1*), and protein kinase cAMP-dependent type II regulatory subunit alpha (*PRKAR2A*) genes may be involved in IMF deposition. Notably, porcine *SCD* was reported to take part in the regulation and biosynthesis process of unsaturated fatty acids [43,44], and its polymorphisms were associated with fatty acid composition in pigs using genome-wide association studies [45]. Moreover, previous studies reported that the expression level of *SCD* is positively correlated with monounsaturated fatty acid in Duroc × Shanzhu commercial crossbreed [16], and mRNA and protein expression levels of *SCD* are significantly higher in fatty Wujin pigs with high IMF content than that in lean Landrace pigs with low IMF content [46]. In the present study, *SCD* expression level also showed a significant difference between the H and L IMF content groups, suggesting that it is an important candidate gene affecting IMF deposition.

Single nucleotide polymorphisms in the other DEGs associated with IMF content trait, including myosin, heavy chain 4 (*MYH4*), and cardiomyopathy associated 5 (*CMYA5*), have been reported in previous studies. Luo et al. performed GWAS of meat quality traits in pigs, and reported that the SNP (ALGA0067099) located at the *MYH4* intron was significantly associated with IMF content [47]. *CMYA5*, named myospryn, belongs to the tripartite motif superfamily of proteins, and is highly expressed in the skeletal muscle and heart [48,49]. The SNP (A383C) of the *CMYA5* gene is significantly correlated with meat quality traits, including drip loss and IMF in pigs [50]. Therefore, these DEGs act as candidates, and their SNPs are useful as genetic markers for IMF content. The DEG *ALDOC,* mapped to the chromosome 12 in pigs [51], activates Wnt pathway signaling by binding with GSK-3β [52]. Recent research found that ALDOC is differently expressed protein between Chinese native breeds and introduced Western breeds, identified using isobaric tag for relative and absolute quantitation (iTRAQ) in longissimus dorsi muscle [53].

The PPAR signaling pathway was significantly enriched in this study, and previous studies indicated that it is a key regulator of lipid metabolism [54]. Therefore, the PPAR signaling pathway is involved in IMF metabolism. Interestingly, the differentially expressed gene *PLIN1*, a lipid droplet coat protein, was included in this pathway. *PLIN1* belongs to the perilipin family proteins, which share highly conserved N-terminal sequences (PAT domain) and a common affinity for intracellular neutral lipid storage droplets [55]. The *PLIN1* gene is located on chromosome 15 in humans [56], while it is localized to chromosome 7 in pigs. In our study, the *PLIN1* gene was also highly expressed in the adipose tissue, and was much lower in other tissues. Previous studies also demonstrated that *PLIN1* is highly expressed in adipocytes and adipose tissues in many species, including mice and humans [57]. *PLIN1*-mediated LD formation can positively regulate sterol regulatory element-binding protein-1 (SREBP-1) activation during adipogenesis, resulting in TG accumulation [58]. Nuclear receptor peroxisome proliferator-activated receptor-γ (PPARγ) enhances *PLIN1* expression through DNA demethylation on PPAR-response elements of *PLIN1* gene promoter upon differentiation [59]. Our results show that the mRNA and protein levels of *PLIN1* were significantly increased in adipocytes after induction differentiation, suggesting that *PLIN1* is involved in the process of porcine adipogenesis. *PLIN1*-null mice had higher intake and normal body weight, while they had less body fat compared with wild-type mice [60]. In our study, the *PLIN1* expression level in the H IMF group was significantly higher than in the L IMF group in pigs. The results indicate that *PLIN1* may promote IMF content and deposition. 

We confirmed that *PLIN1* localizes to lipid droplets in porcine adipocytes, which is consistent with previous studies in human [61] and mouse [35] adipocytes. The results indicate that *PLIN1* is an adipocyte-specific lipid droplet-association protein in porcine adipocytes. Furthermore, *PLIN1* also localizes to adipocytes of porcine longissimus muscle in the study and a similar result is found in semimembranosus muscle [62]. These results suggest that *PLIN1* functions as a lipid droplet-associated protein to regulate IMF deposition. Previous studies have shown that comparative gene identification-58 (CGI-58) binds to *PLIN1* on lipid droplets under basal conditions in adipocytes, preventing interaction with adipose triglyceride lipase (ATGL) [63,64,65,66]. However, in times of energy deficit, CGI-58 can bind and activate ATGL, which catalyzes TG lipolysis [67]. Our data suggest that TG level was significantly decreased in the *PLIN1* knockdown group compared to the control. Therefore, *PLIN1* may increase the TG content of porcine adipocytes by inhibiting TG lipolysis. PLIN1 interacts with CIDEC to promote lipid droplet formation in 3T3-L1 pre-adipocytes [35] and human adipocytes [61]. Intriguingly, we found that the *CIDEC* mRNA expression level was significantly higher in the H IMF group than the L IMF group. These results suggest that *PLIN1* may promote larger lipid droplets formation in the H IMF group by interacting with *CIDEC,* which increased the TG content in the H IMF group.

## 5. Conclusions

In summary, we used RNA-Seq to perform the comparative transcriptome analysis of porcine longissimus dorsi muscle with extreme phenotypes for IMF content in the study. The DEGs identified in our research provide a foundation for elucidating the molecular mechanisms of IMF deposition. Furthermore, our results provide new insights into *PLIN1* influencing the porcine IMF trait.

## Figures and Tables

**Table 1:** Summary statistics of intramuscular fat (IMF) content in the tested samples.

Group	N	IMF (%)	Carcass Weight (kg)
H2	3	3.45 ± 0.12 **	80.60 ± 1.03
L2	3	1.49 ± 0.20	81.13 ± 2.84
H3	3	4.07 ± 0.14 **	80.80 ± 4.24
L3	3	1.71 ± 0.15	83.27 ± 2.33
H5	3	5.13 ± 0.39 **	88.20 ± 1.01
L5	3	1.67 ± 0.12	86.80 ± 3.41

** indicates significant differences between H2 and L2, H3 and L3, H5 and L5 (*p* < 0.01).

**Table 2:** Summary of RNA sequencing (RNA-Seq) data for each sample.

Sample	H2	H3	H5	L2	L3	L5
Total Raw Reads	48,996,194	48,996,096	48,995,932	48,996,332	48,996,238	47,362,614
Total Raw Bases (Gbp)	7.35	7.35	7.35	7.35	7.35	7.10
Total Clean Reads	44,642,506	45,131,966	45,195,292	44,386,936	45,247,560	44,222,498
Total Clean Bases (Gbp)	6.70	6.77	6.78	6.66	6.79	6.63
Clean Reads Q20 (%)	93.05	93.58	95.48	93.46	93.73	95.19
Clean Reads Ratio (%)	91.11	92.11	92.24	90.59	92.35	93.37
GC Content (%)	54.58	54.76	54.79	54.29	54.83	55.05
Total Mapped Reads	27,978,290	29,541,624	30,029,138	28,086,076	29,395,506	29,159,950
Total Mapped Ratio (%)	62.67	65.46	66.44	63.28	64.97	65.94
Unique Mapped Reads	25,460,864	26,757,008	27,174,006	25,570,424	26,637,424	26,461,690
Unique Mapped Ratio (%)	57.03	59.29	60.13	57.61	58.87	59.84
Multiple Mapped Reads	2,517,426	2,784,616	2,855,132	2,515,652	2,758,082	2,698,260
Multiple Mapped Ratio (%)	5.64	6.17	6.32	5.67	6.1	6.1

## Supplementary Materials

The following are available online at http://www.mdpi.com/2073-4425/9/4/194/s1; Figure S1: Graphical abstract of experimental design; Table S1: Primers used in this study; Table S2: Statistical analysis of IMF content (%) of 279 individuals; Table S3: Genes expression level in all samples; Table S4: Summary of DEGs; Table S5: List of GO term enrichment of DEGs; Table S6: List of pathway enrichment of DEGs.

## References

[1] Hocquette J.F., Gondret F., Baeza E., Medale F., Jurie C., Pethick D.W. (2010). Intramuscular fat content in meat-producing animals: Development, genetic and nutritional control, and identification of putative markers. Animal.

[2] Li X., Huang K., Chen F., Li W., Sun S., Shi X.E., Yang G. (2016). Verification of suitable and reliable reference genes for quantitative real-time PCR during adipogenic differentiation in porcine intramuscular stromal-vascular cells. Animal.

[3] Sanchez M.P., Iannuccelli N., Basso B., Bidanel J.P., Billon Y., Gandemer G., Gilbert H., Larzul C., Legault C., Riquet J. (2007). Identification of QTL with effects on intramuscular fat content and fatty acid composition in a Duroc x Large White cross. BMC Genet..

[4] Jeremiah L.E., Dugan M.E.R., Aalhus J.L., Gibson L.L. (2003). Assessment of the chemical and cooking properties of the major beef muscles and muscle groups. Meat Sci..

[5] Jeong D.W., Choi Y.M., Lee S.H., Choe J.H., Hong K.C., Park H.C., Kim B.C. (2010). Correlations of trained panel sensory values of cooked pork with fatty acid composition, muscle fiber type, and pork quality characteristics in Berkshire pigs. Meat Sci..

[6] Anderson F., Pannier L., Pethick D.W., Gardner G.E. (2015). Intramuscular fat in lamb muscle and the impact of selection for improved carcass lean meat yield. Animal.

[7] Newcom D.W., Baas T.J., Schwab C.R., Stalder K.J. (2005). Genetic and phenotypic relationships between individual subcutaneous backfat layers and percentage of longissimus intramuscular fat in Duroc swine. J. Anim. Sci..

[8] Sheard P.R., Nute G.R., Richardson R.I., Wood J.D. (2005). Effects of breed and marination on the sensory attributes of pork from Large White and Hampshire-sired pigs. Meat Sci..

[9] Gjerlaug-Enger E., Aass L., Odegard J., Vangen O. (2010). Genetic parameters of meat quality traits in two pig breeds measured by rapid methods. Animal.

[10] Alonso V., Campo M.D., Espanol S., Roncales P., Beltran J.A. (2009). Effect of crossbreeding and gender on meat quality and fatty acid composition in pork. Meat Sci..

[11] Hamill R.M., McBryan J., McGee C., Mullen A.M., Sweeney T., Talbot A., Cairns M.T., Davey G.C. (2012). Functional analysis of muscle gene expression profiles associated with tenderness and intramuscular fat content in pork. Meat Sci..

[12] Ramayo-Caldas Y., Fortes M.R., Hudson N.J., Porto-Neto L.R., Bolormaa S., Barendse W., Kelly M., Moore S.S., Goddard M.E., Lehnert S.A. (2014). A marker-derived gene network reveals the regulatory role of *PPARGC1A*, *HNF4G*, and *FOXP3* in intramuscular fat deposition of beef cattle. J. Anim. Sci..

[13] Rohrer G.A., Thallman R.M., Shackelford S., Wheeler T., Koohmaraie M. (2006). A genome scan for loci affecting pork quality in a Duroc-Landrace F population. Anim. Genet..

[14] Harlizius B., Rattink A.P., de Koning D.J., Faivre M., Joosten R.G., van Arendonk J.A., Groenen M.A. (2000). The X chromosome harbors quantitative trait loci for backfat thickness and intramuscular fat content in pigs. Mamm. Genome.

[15] Sato S., Ohnishi C., Kikuchi T., Kohira K., Egawa S., Terai S., Nakamura T., Arata S., Komatsuda A., Uemoto Y. (2014). Evaluation of quantitative trait loci affecting intramuscular fat and reproductive traits in pigs using marker-assisted introgression. Anim. Genet..

[16] Wang W., Xue W., Jin B., Zhang X., Ma F., Xu X. (2013). Candidate gene expression affects intramuscular fat content and fatty acid composition in pigs. J. Appl. Genet..

[17] Hu Z.L., Park C.A., Reecy J.M. (2016). Developmental progress and current status of the Animal QTLdb. Nucleic Acids Res..

[18] Wu T., Zhang Z., Yuan Z., Lo L.J., Chen J., Wang Y., Peng J. (2013). Distinctive genes determine different intramuscular fat and muscle fiber ratios of the longissimus dorsi muscles in Jinhua and Landrace pigs. PLoS ONE.

[19] Ma J., Yang J., Zhou L., Ren J., Liu X., Zhang H., Yang B., Zhang Z., Ma H., Xie X. (2014). A splice mutation in the *PHKG1* gene causes high glycogen content and low meat quality in pig skeletal muscle. PLoS Genet..

[20] Hamill R.M., Aslan O., Mullen A.M., O’Doherty J.V., McBryan J., Morris D.G., Sweeney T. (2013). Transcriptome analysis of porcine *M. semimembranosus* divergent in intramuscular fat as a consequence of dietary protein restriction. BMC Genom..

[21] Supakankul P., Mekchay S. (2016). Association of *NLK* polymorphisms with intramuscular fat content and fatty acid composition traits in pigs. Meat Sci..

[22] Kim D., Langmead B., Salzberg S.L. (2015). HISAT: A fast spliced aligner with low memory requirements. Nat. Methods.

[23] Pertea M., Pertea G.M., Antonescu C.M., Chang T.C., Mendell J.T., Salzberg S.L. (2015). StringTie enables improved reconstruction of a transcriptome from RNA-Seq reads. Nat. Biotechnol..

[24] Trapnell C., Roberts A., Goff L., Pertea G., Kim D., Kelley D.R., Pimentel H., Salzberg S.L., Rinn J.L., Pachter L. (2012). Differential gene and transcript expression analysis of RNA-Seq experiments with TopHat and Cufflinks. Nat. Protoc..

[25] Kong L., Zhang Y., Ye Z.Q., Liu X.Q., Zhao S.Q., Wei L., Gao G. (2007). CPC: Assess the protein-coding potential of transcripts using sequence features and support vector machine. Nucleic Acids Res..

[26] Langmead B., Salzberg S.L. (2012). Fast gapped-read alignment with Bowtie 2. Nat. Methods.

[27] Li B., Dewey C.N. (2011). RSEM: Accurate transcript quantification from RNA-Seq data with or without a reference genome. BMC Bioinform..

[28] Tarazona S., Garcia-Alcalde F., Dopazo J., Ferrer A., Conesa A. (2011). Differential expression in RNA-Seq: A matter of depth. Genome Res..

[29] Conesa A., Gotz S., Garcia-Gomez J.M., Terol J., Talon M., Robles M. (2005). Blast2GO: A universal tool for annotation, visualization and analysis in functional genomics research. Bioinformatics.

[30] Wu W., Zhang J., Zhao C., Sun Y., Pang W., Yang G. (2017). CTRP6 regulates porcine adipocyte proliferation and differentiation by the AdipoR1/MAPK signaling pathway. J. Agric. Food Chem..

[31] Untergasser A., Cutcutache I., Koressaar T., Ye J., Faircloth B.C., Remm M., Rozen S.G. (2012). Primer3—New capabilities and interfaces. Nucleic Acids Res..

[32] Koressaar T., Remm M. (2007). Enhancements and modifications of primer design program Primer3. Bioinformatics.

[33] Livak K.J., Schmittgen T.D. (2001). Analysis of relative gene expression data using real-time quantitative PCR and the 2^−ΔΔ*C*T^ method. Methods.

[34] Cristancho A.G., Lazar M.A. (2011). Forming functional fat: A growing understanding of adipocyte differentiation. Nat. Rev. Mol. Cell Biol..

[35] Sun Z., Gong J., Wu H., Xu W., Wu L., Xu D., Gao J., Wu J.W., Yang H., Yang M. (2013). Perilipin1 promotes unilocular lipid droplet formation through the activation of Fsp27 in adipocytes. Nat. Commun..

[36] Huff-Lonergan E., Baas T.J., Malek M., Dekkers J.C.M., Prusa K., Rothschild M.F. (2002). Correlations among selected pork quality traits. J. Anim. Sci..

[37] Zhu J., Shi X., Lu H., Xia B., Li Y., Li X., Zhang Q., Yang G. (2016). RNA-Seq transcriptome analysis of extensor digitorum longus and soleus muscles in large white pigs. Mol. Genet. Genom..

[38] Li B., Dong C., Li P., Ren Z., Wang H., Yu F., Ning C., Liu K., Wei W., Huang R. (2016). Identification of candidate genes associated with porcine meat color traits by genome-wide transcriptome analysis. Sci. Rep..

[39] Maier T., Leibundgut M., Ban N. (2008). The crystal structure of a mammalian fatty acid synthase. Science.

[40] Menendez J.A., Lupu R. (2007). Fatty acid synthase and the lipogenic phenotype in cancer pathogenesis. Nat. Rev. Cancer.

[41] Jeong J., Kwon E.G., Im S.K., Seo K.S., Baik M. (2012). Expression of fat deposition and fat removal genes is associated with intramuscular fat content in longissimus dorsi muscle of Korean cattle steers. J. Anim. Sci..

[42] Zhu B., Niu H., Zhang W., Wang Z., Liang Y., Guan L., Guo P., Chen Y., Zhang L., Guo Y. (2017). Genome wide association study and genomic prediction for fatty acid composition in Chinese Simmental beef cattle using high density SNP array. BMC Genom..

[43] Zappaterra M., Deserti M., Mazza R., Braglia S., Zambonelli P., Davoli R. (2016). A gene and protein expression study on four porcine genes related to intramuscular fat deposition. Meat Sci..

[44] Ntambi J.M. (1995). The regulation of stearoyl-CoA desaturase (SCD). Prog. Lipid Res..

[45] Zhang W.C., Zhang J.J., Cui L.L., Ma J.W., Chen C.Y., Ai H.S., Xie X.H., Li L., Xiao S.J., Huang L.S. (2016). Genetic architecture of fatty acid composition in the longissimus dorsi muscle revealed by genome-wide association studies on diverse pig populations. Genet. Sel. Evol..

[46] Zhao S.M., Ren L.J., Chen L., Zhang X., Cheng M.L., Li W.Z., Zhang Y.Y., Gao S.Z. (2009). Differential expression of lipid metabolism related genes in porcine muscle tissue leading to different intramuscular fat deposition. Lipids.

[47] Luo W., Cheng D., Chen S., Wang L., Li Y., Ma X., Song X., Liu X., Li W., Liang J. (2012). Genome-wide association analysis of meat quality traits in a porcine Large White x Minzhu intercross population. Int. J. Biol. Sci..

[48] Durham J.T., Brand O.M., Arnold M., Reynolds J.G., Muthukumar L., Weiler H., Richardson J.A., Naya F.J. (2006). Myospryn is a direct transcriptional target for MEF2A that encodes a striated muscle, α-actinin-interacting, costamere-localized protein. J. Biol. Chem..

[49] Chen X., Lee G., Maher B.S., Fanous A.H., Chen J., Zhao Z., Guo A., van den Oord E., Sullivan P.F., Shi J. (2011). GWA study data mining and independent replication identify cardiomyopathy-associated 5 (*CMYA5*) as a risk gene for schizophrenia. Mol. Psychiatry.

[50] Xu X., Xu X., Yin Q., Sun L., Liu B., Wang Y. (2011). The molecular characterization and associations of porcine cardiomyopathy associated 5 (*CMYA5*) gene with carcass trait and meat quality. Mol. Biol. Rep..

[51] Lin L., Liu B., Yu M., Yerle M., Fan B., Yang J., Li K. (2004). Radiation hybrid mapping of the pig *ALDOA*, *ALDOB* and *ALDOC* genes to SSC3, SSC1 and SSC12. Anim. Genet..

[52] Caspi M., Perry G., Skalka N., Meisel S., Firsow A., Amit M., Rosin-Arbesfeld R. (2014). Aldolase positively regulates of the canonical Wnt signaling pathway. Mol. Cancer.

[53] Wang Z., Shang P., Li Q., Wang L., Chamba Y., Zhang B., Zhang H., Wu C. (2017). iTRAQ-based proteomic analysis reveals key proteins affecting muscle growth and lipid deposition in pigs. Sci. Rep..

[54] Takada I., Kouzmenko A.P., Kato S. (2009). Wnt and PPARγ signaling in osteoblastogenesis and adipogenesis. Nat. Rev. Rheumatol..

[55] Kimmel A.R., Brasaemle D.L., McAndrews-Hill M., Sztalryd C., Londos C. (2010). Adoption of PERILIPIN as a unifying nomenclature for the mammalian PAT-family of intracellular lipid storage droplet proteins. J. Lipid Res..

[56] Kimmel A.R., Sztalryd C. (2016). The perilipins: Major cytosolic lipid droplet-associated proteins and their roles in cellular lipid storage, mobilization, and systemic homeostasis. Annu. Rev. Nutr..

[57] Greenberg A.S., Egan J.J., Wek S.A., Moos M.C., Londos C., Kimmel A.R. (1993). Isolation of cDNAs for perilipins A and B: Sequence and expression of lipid droplet-associated proteins of adipocytes. Proc. Natl. Acad. Sci. USA.

[58] Takahashi Y., Shinoda A., Furuya N., Harada E., Arimura N., Ichi I., Fujiwara Y., Inoue J., Sato R. (2013). Perilipin-mediated lipid droplet formation in adipocytes promotes sterol regulatory element-binding protein-1 processing and triacylglyceride accumulation. PLoS ONE.

[59] Fujiki K., Shinoda A., Kano F., Sato R., Shirahige K., Murata M. (2013). PPARγ-induced PARylation promotes local DNA demethylation by production of 5-hydroxymethylcytosine. Nat. Commun..

[60] Martinez-Botas J., Anderson J.B., Tessier D., Lapillonne A., Chang B.H., Quast M.J., Gorenstein D., Chen K.H., Chan L. (2000). Absence of perilipin results in leanness and reverses obesity in *Lepr^db/db^* mice. Nat. Genet..

[61] Grahn T.H., Zhang Y., Lee M.J., Sommer A.G., Mostoslavsky G., Fried S.K., Greenberg A.S., Puri V. (2013). FSP27 and PLIN1 interaction promotes the formation of large lipid droplets in human adipocytes. Biochem. Biophys. Res. Commun..

[62] Gandolfi G., Mazzoni M., Zambonelli P., Lalatta-Costerbosa G., Tronca A., Russo V., Davoli R. (2011). Perilipin 1 and perilipin 2 protein localization and gene expression study in skeletal muscles of European cross-breed pigs with different intramuscular fat contents. Meat Sci..

[63] Sahu-Osen A., Montero-Moran G., Schittmayer M., Fritz K., Dinh A., Chang Y.F., McMahon D., Boeszoermenyi A., Cornaciu I., Russell D. (2015). CGI-58/ABHD5 is phosphorylated on Ser239 by protein kinase A: Control of subcellular localization. J. Lipid Res..

[64] Subramanian V., Rothenberg A., Gomez C., Cohen A.W., Garcia A., Bhattacharyya S., Shapiro L., Dolios G., Wang R., Lisanti M.P. (2004). Perilipin A mediates the reversible binding of CGI-58 to lipid droplets in 3T3-L1 adipocytes. J. Biol. Chem..

[65] Yamaguchi T., Omatsu N., Matsushita S., Osumi T. (2004). CGI-58 interacts with perilipin and is localized to lipid droplets—Possible involvement of CGI-58 mislocalization in Chanarin-Dorfman syndrome. J. Biol. Chem..

[66] Miyoshi H., Perfield J.W., Souza S.C., Shen W.J., Zhang H.H., Stancheva Z.S., Kraemer F.B., Obin M.S., Greenberg A.S. (2007). Control of adipose triglyceride lipase action by serine 517 of perilipin A globally regulates protein kinase A-stimulated lipolysis in adipocytes. J. Biol. Chem..

[67] Patel S., Yang W., Kozusko K., Saudek V., Savage D.B. (2014). Perilipins 2 and 3 lack a carboxy-terminal domain present in perilipin 1 involved in sequestering ABHD5 and suppressing basal lipolysis. Proc. Natl. Acad. Sci. USA.

