# Supplementary material for: A Key Gene, PLIN1, Can Affect Porcine Intramuscular Fat Content Based on Transcriptome Analysis
Source: Genes (Basel). 2018 Apr 4;9(4):194. doi: 10.3390/genes9040194 (PMC5924536; doi:10.3390/genes9040194)
Supplement: Supplementary file 1 [file genes-09-00194-s001.zip › Supplementary File(s)/Table S2.docx]

**Table S2.** Statistical analysis of IMF content (%) of 279 individuals.

| Group | Number | Mean | SD | Maximum | Minimum | CV (%) |
| --- | --- | --- | --- | --- | --- | --- |
| 1 | 25 | 2.63 | 0.79 | 4.48 | 1.28 | 30.04 |
| 2 | 25 | 2.52 | 0.82 | 4.92 | 1.12 | 32.54 |
| 3 | 25 | 2.98 | 1.13 | 5.68 | 1.38 | 37.92 |
| 4 | 25 | 2.53 | 0.56 | 3.48 | 1.35 | 22.13 |
| 5 | 25 | 2.91 | 0.84 | 4.5 | 1.42 | 28.87 |
| 6 | 25 | 3.19 | 1.13 | 5.68 | 1.54 | 35.42 |
| 7 | 25 | 2.71 | 0.94 | 4.91 | 1.23 | 34.69 |
| 8 | 25 | 2.59 | 0.98 | 6.56 | 1.58 | 37.84 |
| 9 | 25 | 2.15 | 0.36 | 2.87 | 1.49 | 16.74 |
| 10 | 23 | 2.78 | 1.05 | 5.51 | 1.19 | 37.77 |
| 11 | 31 | 2.17 | 0.77 | 3.83 | 1.09 | 35.48 |
| Total | 279 | 2.64 | 0.91 | 6.56 | 1.09 | 34.47 |

SD: Standard deviation

CV: Coefficient of variation
