# Supplementary figures and images for: A Key Gene, PLIN1, Can Affect Porcine Intramuscular Fat Content Based on Transcriptome Analysis
Source: Genes (Basel). 2018 Apr 4;9(4):194. doi: 10.3390/genes9040194 (PMC5924536; doi:10.3390/genes9040194)

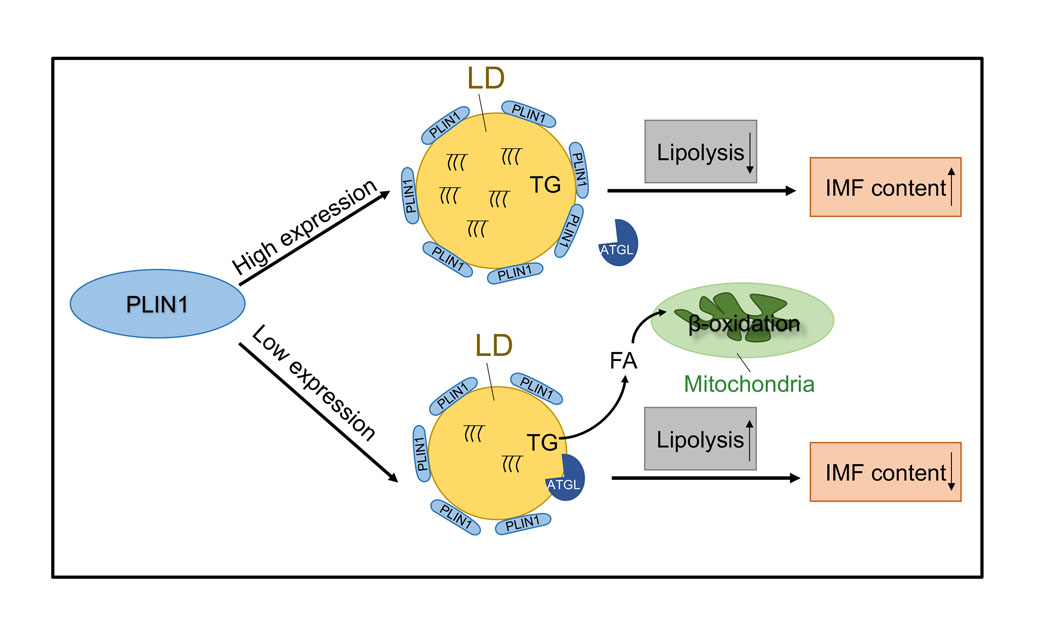

Supplement: Supplementary file 1 [file genes-09-00194-s001.zip › Supplementary File(s)/genes-277628-Graphical abstract.jpg]
