# Supplementary material for: A Key Gene, PLIN1, Can Affect Porcine Intramuscular Fat Content Based on Transcriptome Analysis
Source: Genes (Basel). 2018 Apr 4;9(4):194. doi: 10.3390/genes9040194 (PMC5924536; doi:10.3390/genes9040194)
Supplement: Supplementary file 1 [file genes-09-00194-s001.zip › Supplementary File(s)/ Table S1.doc]

**Table S1.** Primers used in this study.

| Gene name | Primer sequences | Product size | Annealing temperature |
| --- | --- | --- | --- |
| COQ10A | F: GGCTGAGGCTGACTTATCT  R: TTCTTACACCAGGGTACAAAC | 171 bp | 60°C |
| DCTN2 | F: GCCTTGTCACTTACGAACT  R: TTATGCTTGGCAATCTCA | 283 bp | 60°C |
| CIDEC | F: ACGGTGCCTACTCCCTTTCCT  R: TTTGCCCTTGGTGGGTTGTTC | 179 bp | 60°C |
| FASN | F: TCCTCCGCTCGCTCCATCCT  R: CCATCGTGTTCGCCTGCTTG | 289 bp | 60°C |
| SCD | F: GCCTACTATCTGCTGAGTGC  R: TCTCGGGCCCATTCATAAAC | 152 bp | 60°C |
| PLIN1 | F: GCAATGCTTACGAGAAGGGC  R: ATACTGGAGGGCCGGTATCT | 158 bp | 60°C |
| ALDOC | F: TTGCGGATCTGAACCTGACCTG  R: CCTTGCCTGGGGCTACAATC | 163 bp | 60°C |
| PTX3 | F: CCCGGCAACACCAATGAAAC  R: CGTTGACCCACAAGGCTACA | 269 bp | 60°C |
| NDUFA6 | F: CCTCGGTGAAGCCCATTT  R: AACCACCCTGGCGTCTGT | 194 bp | 60°C |
| COMMD5 | F: AGGACCTGGTTGTGGACTTG  R: CGTCCACCCTCCACCTAAAG | 123 bp | 60°C |
| GAPDH | F: ATCACTGCCACCCAGAAGACT  R: CATGCCAGTGAGCTTCCCGTT | 153 bp | 60°C |
